# Intake of Pistachios as a Nighttime Snack Has Similar Effects on Short- and Longer-Term Glycemic Control Compared with Education to Consume 1–2 Carbohydrate Exchanges in Adults with Prediabetes: A 12-Wk Randomized Crossover Trial

**DOI:** 10.1016/j.tjnut.2024.01.021

**Published:** 2024-01-24

**Authors:** Terrence M Riley, Penny M Kris-Etherton, Tricia L Hart, Kristina S Petersen

**Affiliations:** Department of Nutritional Sciences, The Pennsylvania State University, University Park, PA, United States

**Keywords:** prediabetes, nutritional intervention, pistachios, carbohydrate exchanges, randomized crossover trial

## Abstract

**Background:**

Nut intake is associated with better glycemic control and lower cardiovascular disease (CVD) risk. It remains unclear if nut intake timing affects glycemic control and CVD risk factors. Intake of pistachios as a nighttime snack may attenuate morning glucose production and lower fasting plasma glucose (FPG).

**Objectives:**

We assessed the effects of a nighttime (after dinner and before bedtime) pistachio snack (57 g/d) on glycemic control markers, vascular health, lipids/lipoproteins, and diet quality compared with education to consume 1–2 carbohydrate (CHO) exchanges (usual care) in individuals with prediabetes.

**Methods:**

A 2-period, randomized crossover trial was conducted. Participants were provided 57 g/d of dry roasted unsalted pistachios (319 kcal; fat 26 g; CHO 16 g; protein 12 g; fiber 6 g) as a nighttime snack or received usual care for 12 wk. Primary (FPG) and secondary outcomes [hemoglobin A1c (HbA1c), insulin, Homeostatic Model Assessment for Insulin Resistance (HOMA-IR), lipids/lipoproteins, vascular health, and Healthy Eating Index-2015 (HEI-2015)] were measured before and after each condition.

**Results:**

A total of 66 participants (50.9 ± 11.6 y, FPG: 106.2 ± 6.4 mg/dL) were randomly assigned, and 51 participants completed the trial. No between-condition differences in FPG {0.9 mg/dL [95% confidence interval (CI): −1.2, 3.1]}, HbA1c, insulin, HOMA-IR, lipids/lipoproteins, blood pressure, or vascular health were observed. The HEI-2015 score was higher after the pistachio condition [6.8 points (95% CI: 1.5, 12.1)] than after usual care driven by higher component scores for seafood and plant proteins [2.0 points (95% CI: 1.0, 2.9)], refined grains [2.3 points (95% CI: 1.1, 3.5)], and the fatty acid ratio [1.7 points (95% CI: 0.0, 3.5)].

**Conclusions:**

In adults with prediabetes, consuming 57 g/d of pistachios as a nighttime snack increased diet quality but had similar effects on glycemic markers, lipids/lipoproteins, blood pressure, and vascular health compared with the usual care comparator. Pistachios may be a healthful alternative to carbohydrate-rich nighttime snacks to increase alignment with Dietary Guidelines for Americans.

This trial was registered at clinicaltrials.gov as NCT04056208.

## Introduction

An estimated 38% of United States adults have prediabetes [1]. Of concern, ∼74% of adults with prediabetes in midlife will develop type 2 diabetes mellitus (T2DM) in their lifetime [2]. Prediabetes is defined by impaired fasting glucose (100–125 mg/dL), elevated hemoglobin A1c (HbA1c) (5.7%–6.4%), or impaired glucose tolerance (140–199 mg/dL) [3]. Impaired fasting glucose in midlife is associated with an ∼50% increase in 30-y relative risk of incident cardiovascular disease (CVD) [4]. Current treatments for prediabetes to prevent or delay the onset of T2DM include intensive lifestyle and behavioral therapies such as sustained weight loss, medical nutrition therapy to normalize dysglycemia, and/or moderate-to-intense physical activity [5]. However, there may be barriers to accessing these intensive lifestyle therapies [6]. Effective low-intensity strategies are needed to improve glycemic control in individuals with prediabetes to prevent or delay the onset of T2DM.

Diet and lifestyle intervention is the first-line therapy for prediabetes to prevent or delay further impairments in glycemic control. A common clinical recommendation for those with impaired fasting glucose is to consume a nighttime snack after dinner but before bedtime because this period has the greatest influence on morning hyperglycemia [7,8]. A common treatment strategy to lower morning hyperglycemia is to consume a snack after dinner but before bedtime [7]. A bedtime snack may reduce the gluconeogenic demand overnight, resulting in lower fasting glucose and attenuation of postprandial glycemia after breakfast [9]. Recommended nighttime snacks usually include carbohydrate (CHO)-containing foods because an even daily distribution of CHOs reduces glycemic excursions [10]. Nighttime CHO intake has been shown to improve morning glucose excursions and daytime glycemic control [9,[11], [12], [13]]. Both the American Diabetes Association (ADA) and the Academy of Nutrition and Dietetics recommend CHO counting, also called “CHO exchanges,” as a method of helping patients track daily CHO consumption [14,15]. However, it is unclear whether nighttime CHO snacking using CHO exchanges is the most effective approach to improve glycemic control.

Consumption of pistachios may be a convenient and healthful approach to reduce morning hyperglycemia. Several recent meta-analyses of randomized controlled trials (RCTs) show that pistachios improve fasting blood glucose, insulin, HbA1c, and insulin resistance measured by HOMA-IR compared with no nut interventions [[16], [17], [18], [19], [20]]. The improvements in glycemic control are likely attributable to the high fiber content of pistachios and their unsaturated to saturated fatty acid ratio, which are known to improve lipids/lipoproteins and vascular health [[21], [22], [23]]. In addition, nuts are recommended as part of healthful dietary patterns [24]. Given the evidence showing improved glycemic control with pistachio intake, intake of pistachios as a nighttime snack may be a convenient and effective approach to improve morning hyperglycemia.

The aim of the present randomized controlled crossover trial was to evaluate the effect of consuming 57 g/d (∼2 oz) of pistachios as a nighttime snack compared with 1–2 CHO exchanges (15–30 g of CHOs), on glycemic control as well as risk factors for CVD and diet quality in adults with impaired fasting plasma glucose (FPG). It was hypothesized that daily consumption of pistachios as a nighttime snack for 12-wk would improve FPG, risk factors for CVD, and diet quality compared with the usual care condition, which included education to consume 1–2 CHO exchanges.

## Methods

### Trial design

A single-blind, 2-period, randomized crossover trial was conducted at the Pennsylvania State University between September 2019 and November 2022. Study activities were suspended in March of 2020 because of COVID-19. Research activities resumed in March of 2021. Diet periods were ∼12 wk in duration and separated by a washout period of ≥4 wk. Eligible individuals were randomly allocated in a 1:1 ratio to 2 randomization sequences using a computer-generated scheme (randomization.com) that contained blocks of 10 sequences. The randomization sequence was generated by an investigator not involved in data collection. The randomization code was held by the staff member delivering the intervention and participants were randomly assigned after baseline testing was scheduled. All other research personnel involved in screening, data collection, and analysis were unaware of the randomization schedule until after trial completion. Blinding of participants was not possible because of the nature of the study conditions. This study is registered at clinicaltrials.gov (identifier: NCT04056208). The study protocol was approved by the Institutional Review Board of the Pennsylvania State University. All participants provided written informed consent.

### Participants

Participants were recruited from State College, Pennsylvania, and surrounding areas. Recruitment involved posting flyers in local businesses and university buildings as well as advertisements in local magazines and circulars. The study was also posted on clinicaltrials.gov, StudyFinder (studyfinder.com), and our research group’s webpage. Interested individuals were telephone screened to assess potential eligibility. A follow-up clinic screening visit was then scheduled at the Pennsylvania State University Clinical Research Center to determine study eligibility. Measures of height, weight, blood pressure (BP), waist circumference, and blood sampling were performed during the clinic screening appointment. Height was measured during the screening appointment without shoes. Participants were weighed with light clothing and without shoes. BP was measured using a validated automated sphygmomanometer 3 times after a 5-min rest [25]; the mean of the last 2 measurements was used to determine eligibility. Nurses collected a fasting blood sample for a complete blood count, blood chemistry, and plasma glucose, which were assayed by a commercial laboratory (Quest Diagnostics). Before the clinic screening, individuals were fasted (no food or drink except water) for ≥12 h and avoided over-the-counter medications and alcohol for 48 h.

Males and females aged 30–75 y with a BMI ≥ 25 and ≤ 45 kg/m^2^ and elevated FPG (≥100 mg/dL and ≤125 mg/dL) measured at screening were eligible. We included individuals with a BMI ≥ 25 and ≤ 45 kg/m^2^ because lifetime risk of type 2 diabetes increases with higher BMI [2]. After the COVID-19 shutdown of research activities in March 2020, research was resumed in March 2021 after University officials (Dean of the College of Health and Human Development, Campus Chancellors, the Senior Vice President for Research) and the institutional review board approved research activities contingent upon modifying the age inclusion criterion to 30–65 y. Age of inclusion was modified because available data at that time showed higher risk of adverse outcomes and severe COVID-19 for individuals aged >65 y and vaccines were unavailable at the time. Therefore, participation in this research for participants aged >65 y, including those randomly assigned before 2021, was deemed as beyond an acceptable risk level and these participants were no longer eligible for the study. This was the only eligibility criterion modified. Participants who commenced diet period 1 before the COVID-19 shutdown were rescreened after the restart of study activities in 2021. Participants who completed diet period 1 before the COVID-19 shutdown aged ≤65 y started diet period 2 after the restart of study activities in 2021.

The exclusion criteria included a diabetes diagnosis (any type); systolic blood pressure (SBP) >160 mm Hg or diastolic blood pressure (DBP) >100 mm Hg; use of antihypertensive, lipid-lowering or glucose-lowering drugs; or use of steroids or antibiotics in the previous month. In addition, eligible individuals did not have cardiovascular, liver or kidney disease, autoimmune disorders, or inflammatory conditions such as gastrointestinal disorders or rheumatoid arthritis. Other exclusion criteria were use of supplements (psyllium, fish oil, soy lecithin, and phytoestrogens) or botanicals and not willing to discontinue use for the duration of the study; pregnancy, lactation, or plans to become pregnant or have given birth in the past year; weight loss of ≥10% of body weight within the 6 mo before enrolling in the study; or smoking or use of any tobacco products in past 6 mo. In addition, shiftwork, an inability to consume a snack in the evening, allergy/intolerance/sensitivity to pistachios, consumption of >14 alcoholic drinks per week or not willing to avoid alcohol consumption for 48 h before test visits were exclusion criteria. Individuals who donated blood within the previous 8 wk, or those unwilling to refrain from donating blood during the study were also ineligible.

### Intervention and comparator

During the pistachio condition, participants were provided with 57 g/d of dry roasted unsalted pistachios [324 kcal (fat: 26 g; CHOs: 16.04 g; protein: 11.9 g; saturated fat: 3.2 g; monounsaturated fat: 13.9 g; polyunsaturated fat: 7.54 g; fiber: g; sodium: 3.4 mg)] with education to consume them as a nighttime snack. During the control condition (usual care), participants were given education to consume 1–2 CHO exchanges (15–30 g of CHOs) as a nighttime snack and provided with a gift card to a local grocery store, in approximately equal value to the pistachios, to purchase the snacks. Thus, the monetary value of the conditions was matched.

During both conditions a handout was provided including information about when to consume study foods, how much to consume, and to avoid consuming other calorie-containing foods or beverages during and after the nighttime snack. The handout for the control condition included information about CHO exchanges, CHO-containing food sources, and the quantity of different foods (for example, whole wheat bread, different types of crackers including whole wheat, plain popcorn, plain pretzels) that met the 1–2 CHO exchange criteria, which was adapted from an ADA resource [26]. This comparator was selected because CHO-counting education is among the recommended clinical approaches for achieving greater time in target glucose range and was not expected to negatively impact glucose control in those at risk of T2DM [5,15]. The education was conducted by the metabolic kitchen manager. For both conditions, participants were instructed to consume the snacks after dinner but before bedtime and avoid any other calorie-containing food or drink in the evening. Participants were asked to avoid any other tree nuts, peanuts, including nut butters, or additional pistachios not provided by the study team throughout the study.

During both conditions, participants met with the metabolic kitchen manager to receive pistachios or a gift card (depending on the condition) every 2 wk. The time spent with the kitchen manager was matched for the conditions. The monthly supply of pistachios was provided in daily 57 g portions. A single $30 gift card was provided for each month of the usual care condition. In addition, adherence checks were conducted for both conditions, and re-education was provided when needed.

Adherence was assessed using a daily adherence checklist completed by the participants at home. The checklist included questions about consumption of the study foods, if the study foods were consumed after dinner, a description of the snack (during the usual care condition), if other calorie-containing foods/beverages were consumed after intake of the study nighttime snack, if other peanuts/tree nuts were consumed, if changes in health or usual exercise habits occurred, and if any nonhabitual medications or supplements were taken. After the restart of the study after the COVID-19 shutdown, the frequency of study visits was reduced to monthly for both conditions to limit in-person contact. Adherence checks for both conditions were conducted biweekly by telephone.

Calculation of adherence to each condition was performed using the adherence checklists completed biweekly throughout the study. For the pistachio condition, the number of days the pistachios were consumed as directed (that is, as a nighttime snack, without intake of any other food or drinks after dinner) was divided by the number of days in the diet period. For the usual care condition, the number of days CHO-containing foods were consumed as directed (that is, as a nighttime snack, without intake of any other food or drinks after dinner) was divided by the total number of days in the diet period.

### Outcome assessment

Outcomes were measured on 2 consecutive days at the beginning and the end of each diet period (8 visits total). Participants were instructed to fast for 12 h before testing, avoid strenuous physical activity for 12 h, and refrain from drinking alcohol or taking over-the-counter medications for 48 h before each visit. Weight was measured on both days with a calibrated electronic scale while participants were wearing light clothing and no shoes. Blood was drawn at both visits to measure glucose, lipids and lipoproteins, and insulin. HbA1c was measured on 1 day at the beginning and the end of each diet period. Vascular testing was performed on 1 of the 2 consecutive test days at each timepoint.

### Blood processing and assay methods

Blood samples were drawn into 3 different collection vacutainers: serum separator, EDTA, and sodium fluoride (NaF)/potassium oxalate (KOx). Immediately after blood collection, tubes with NaF/KOx were inverted and centrifuged (1590 ± 90 × *g*) at room temperature for 15 min. The plasma supernatant was stored. EDTA tubes containing whole blood were inverted 8 times before aliquot storage. Serum separator tubes were left at room temperature to clot for 30 min then centrifuged (1590 ± 90 × *g*) for 15 min. All samples were frozen at −80°C upon collection for batch analysis at the end of the study. Glucose was measured in plasma samples. HbA1c was measured in whole blood. Serum total cholesterol, LDL-C, HDL-C, and insulin were measured from serum separator tubes. Samples were assayed at the Pennsylvania State University Biomarker Core Lab using a Cobas c311 chemistry analyzer (Roche Diagnostics) according to the manufacturer’s instructions. HOMA-IR was calculated using the following formula: fasting plasma insulin (μU/mL) × FPG (mg/dL)/405 [27]. Interassay coefficient of variation is estimated at <5% for all tests according to the manufacturers insert.

### Vascular testing methods

Vascular measures were performed with a SphygmoCor XCEL (AtCor Medical). After a 5-min rest, peripheral and central BP were measured in triplicate with participants resting in the seated position. Peripheral BP and radial artery waveforms were measured with a cuff placed on the left arm. Central BP was calculated from the measured peripheral BP and radial artery pressure waveform using a validated generalized transfer function. A heart rate of 75 beats/min was used as the adjustment for augmentation index. Immediately after the BP assessment, carotid-femoral pulse wave velocity (PWV) was measured with the SphygmoCor XCEL. Participants lay supine while a BP cuff was placed around the thigh on the femoral artery. A tonometer was placed on the carotid artery. The carotid-femoral waveform was recorded for 10 s. PWV was calculated by dividing the linear distance between the carotid and femoral sites by the transit time. The final 2 measures for each outcome were averaged and used for the analyses.

### Dietary assessment

Dietary intake was assessed by nonrandom, participant-completed 24-h recalls before each diet period (precondition) and in the last week of each diet period (postcondition). Participants were asked to complete a total of four 24-h recalls (that is, 1 before each condition and 1 at each endpoint) throughout the study using the Automated Self-Administered 24-h Dietary Assessment Tool (ASA24) [28]. The diet recall tool was administered as recommended by the National Cancer Institute (NCI) Dietary Assessment Primer [29]. Briefly, participants were provided with a unique username and password to access the ASA24 diet recall tool and an instruction sheet explaining how to complete the recall. Participants were asked to complete the 24-h recall before study appointments. The Healthy Eating Index (HEI)-2015 was used to assess diet quality. The HEI-2020 was recently released and it is exactly the same as HEI-2015, although the index was renamed to reflect the alignment with the 2020–2025 Dietary Guidelines for Americans [30]. The HEI-2015 contains 13 components. Nine are “adequacy” components (whole grains, total fruits, dairy, etc.) and 4 are “moderation” components (saturated fats, sodium, refined grains, and sugars). Consuming more adequacy components increases the HEI-2015 score, whereas lower consumption of moderation components reflects a higher score. The HEI-2015 was calculated using the Statistical Analysis System (SAS) code created by the NCI [31]. Recalls were excluded if reported energy intake was <600 or >4400 kcal/d for females and <650 or >5700 kcal/d for males based on the NCI guidelines for reviewing and cleaning ASA24 data [32].

### Statistical analyses

The study was powered for the primary outcome, FPG. On the basis of prior research examining intake of >28 g/d of pistachios, to detect a 10 mg/dL difference (SD: 23.4 mg/dL) in FPG between conditions, 45 participants were required (80% power; α = 0.05, 2-tailed test) [[33], [34], [35], [36], [37], [38]]. Target enrollment was 59 individuals to ensure 45 subjects completed the protocol (assuming ∼25% drop out rate). All statistical analyses were performed using SAS (version 9.4; SAS Institute Inc.). The normality of the residuals was assessed with univariate analysis (PROC UNIVARIATE). The distribution and normal probability plots (Q–Q plots) were evaluated and for all outcomes; the residual distribution was approximately normal. For outcomes measured on both test days at each timepoint, the average was used for analysis. Descriptive data are presented as means ± SD unless otherwise stated. Within- and between-condition effect estimates are presented as least-squared mean and 95% confidence interval (CI). Between-condition differences in endpoint means for each outcome variable were examined using the mixed-models procedure (PROC MIXED) at a predetermined α level of 0.05. Randomization sequence and condition were modeled as fixed effects. Participant nested within randomization sequence was modeled as a repeated effect to account for the crossover design. Precondition values were included as a covariate. Carryover effects and sex differences were determined by including randomization sequence, sex, and their interaction by condition (that is, sex∗condition, randomization sequence∗condition) in the model as fixed effects. In the primary analysis, the between-condition difference in endpoint mean values for each outcome was assessed with adjustment for the precondition value. Secondary analyses assessed within-condition change from precondition for all outcome variables. The covariance structure was determined using an optimized fit approach (lowest Bayesian information criterion), which varied depending upon the analysis being conducted. Data analyses included all randomly assigned participants with data available at ≥1 timepoint. The mixed-models procedure does not perform listwise deletion preserving the degrees of freedom; therefore, this analytic approach allows inclusion of participants with ≥1 missing data point. Outliers >3 SDs from the sample mean were removed before analysis for primary and secondary outcomes [39,40]. Outliers were removed because the values were not physiologically plausible given individuals were in the fasted state. For all outcomes, <2% of data were removed (Supplemental Table 1). McNemar’s exact chi-squared test (PROC FREQ) was used to determine whether HEI-2015 scores for nighttime snacks differed by condition from the beginning of the trial. Nighttime snacks, identified as foods reported in the 24-h recall consumed after dinner but before bedtime, were categorized based on the mean (≤mean and >mean) HEI-2015 score for nighttime snacks before starting the first diet period.

## Results

**FIGURE 1:**
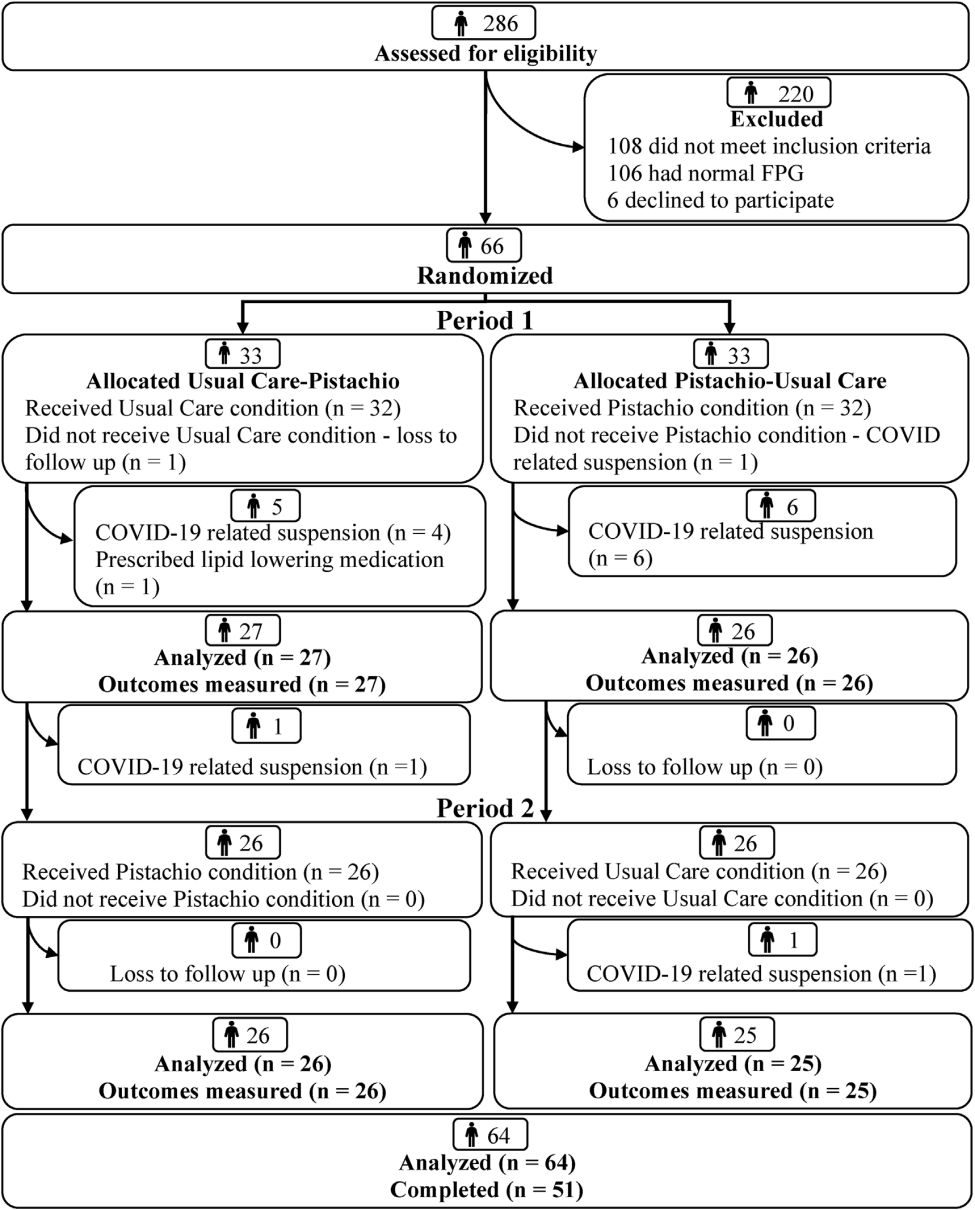
CONSORT flow diagram.

Among the 286 individuals who completed a telephone screening, 66 participants were randomly assigned (Figure 1). Baseline testing was conducted in 64 participants. In total, 51 participants completed the trial. The COVID-19 pandemic resulted in the suspension of human subject’s research at Penn State University after 9 months of study activities. At that time, 23 participants were enrolled. Of these participants, 13 withdrew from the study, and 5 participants restarted diet period 1 and 5 participants restarted diet period 2 when study activities resumed. For the 13 participants who were withdrawn, 5 no longer met the age inclusion criterion (age > 65 y), 5 were lost to follow-up, and 3 did not meet the FPG criterion upon rescreening. After the study resumed after the COVID-19 shutdown, 43 participants were randomly assigned. Of these participants, 1 was lost to follow-up before starting diet period 1 and another was withdrawn from the study before the end of diet period 1 because prescription of a lipid-lowering medication was reported.

Randomly assigned participants (27 females and 39 males) were on average 50.9 ± 11.6 y (mean ± SD) with a BMI of 31.2 ± 4.0 kg/m^2^ and FPG of 106.2 ± 6.4 mg/dL at screening (Table 1). Adherence to the intervention (days consumed as directed/days in the diet period) was 90% during the pistachio condition and 93% during the usual care condition. The most commonly reported snacks consumed by participants on the usual care condition were crackers, popcorn, and pretzels.

**TABLE 1:** Characteristics of participants overall and by randomization sequence at screening^1^

Characteristic	Pistachio→Usual care	Usual care→Pistachio	Total
*n* (% female)	33 ± 30.0	33 ± 48.4	66 ± 40.9
Age (y)	51.9 ± 12.7	51.2 ± 10.1	50.9 ± 11.6
Weight (kg)	91.8 ± 15.0	93.1 ± 16.5	92.5 ± 15.6
Height (m)	1.71 ± 0.09	1.72 ± 0.08	1.72 ± 0.09
BMI (kg/m^2^)	31.1 ± 3.7	31.3 ± 4.4	31.2 ± 4.0
FPG (mg/dL)	106.5 ± 6.8	105.8 ± 6.0	106.2 ± 6.4
WC (males) (cm)	103.6 ± 11.8	106.9 ± 13.0	105.8 ± 12.3
WC (females) (cm)	103.5 ± 8.5	101.3 ± 12.0	101.3 ± 10.3

Abbreviations: FPG, fasting plasma glucose; WC, waist circumference.

1 *n* = 66. Data are mean ± SD unless otherwise stated. Usual care is defined as education to consume 1–2 carbohydrate (CHO) exchanges (15 g–30 g CHOs) as a nighttime snack.

No significant between-condition mean differences (MDs) in FPG, HbA1c, insulin, and HOMA-IR, weight, BMI, lipids/lipoproteins, and vascular health measures (*P* > 0.05) were observed (TABLE 2, TABLE 3).

**TABLE 2:** Within- and between-condition mean differences for glucose control, lipids/lipoproteins, and anthropometrics in adults with prediabetes^1^

Outcome	Study baseline^5^	Pistachio		Within-condition difference^4^	Usual care		Within-condition difference^4^	Between-condition difference^4^
		Pre^2^	Post^3^		Pre^2^	Post^3^		
Glucose (mg/dL)	103.2 ± 8.8	103.1 ± 8.7	102.7 ± 7.5^6^	0.9 (−0.6, 2.5)	102.4 ± 8.7	101.8 ± 8.1	−0.0 (−1.5, 1.5)	0.9 (−1.2, 3.1)
HbA1c (%)	5.4 ± 0.3	5.4 ± 0.3	5.5 ± 0.3	−0.0 (−0.0, 0.0)	5.4 ± 0.2	5.4 ± 0.2	−0.0 (−0.0, 0.0)	0.0 (−0.0, 0.0)
Insulin (μIU/mL)	12.6 ± 7.2	12.9 ± 7.5	13.9 ± 9.1	0.9 (−0.3, 2.2)	12.1 ± 7.5	11.8 ± 7.3	−0.1 (−1.4, 1.1)	1.1 (−0.6, 2.9)
Homeostatic Model Assessment for Insulin Resistance	3.2 ± 2.0	3.3 ± 2.0	3.6 ± 2.5^6^	0.2 (−0.0, 0.6)	3.1 ± 2.1	2.9 ± 1.9	−0.0 (−0.4, 0.2)	0.3 (−0.1, 0.8)
Weight (kg)	93.2 ± 15.8	93.9 ± 16.8	94.8 ± 16.9	0.7 (0.1, 1.4)^8^	93.4 ± 15.7	94.2 ± 16.5	−0.0 (−0.6, 0.6)	0.7 (−0.1, 1.7)
BMI (kg/m^2^)	31.4 ± 4.3	31.6 ± 4.5	31.7 ± 4.5	0.2 (0.0, 0.4)^8^	31.3 ± 4.3	31.5 ± 4.4	0.0 (−0.2, 0.2)	0.2 (−0.0, 0.5)
Total cholesterol (mg/dL)	199.5 ± 33.6	199.7 ± 32.3	196.5 ± 37.4	−3.7 (−8.6, 1.0)	197.5 ± 39.6	198.7 ± 35.1	−0.8 (−5.6, 3.9)	−2.9 (−9.7, 3.9)
LDL-C (mg/dL)	124.9 ± 29.3	124.8 ± 24.9	122.1 ± 28.8	−3.4 (−7.4, 0.5)	122.2 ± 30.7	123.7 ± 28.7	−0.2 (−4.1, 3.7)	−3.0 (−8.6, 2.5)
HDL-C (mg/dL)	51.1 ± 13.5	50.9 ± 13.8	50.0 ± 12.7	−1.3 (−2.4, −0.2)^8^	51.8 ± 13.4	50.9 ± 12.9	−0.7 (−1.8, 0.3)	0.6 (−2.1, 0.9)
TAG (mg/dL)	114.0 ± 51.1	116.5 ± 51.5	116.9 ± 59.3	2.2 (−6.0, 10.5)	107.9 ± 46.8^7^	118.1 ± 57.3	3.0 (−5.3, 11.3)	−0.7 (−12.5, 11.0)

Abbreviations: CI, confidence interval; HbA1c, hemoglobin A1c; TAG, Triacylglycerols.

1 *n* = 64 unless otherwise stated. Statistical analyses were performed with SAS version 9.4 (SAS Institute). The MIXED procedure was used to determine the within- and the between-condition mean difference adjusted for the precondition value. Usual care is defined as education to consume 1–2 carbohydrate exchanges (15–30 g CHOs).

2 Values are arithmetic means ± SD; *n* = 58.

3 Values are arithmetic mean ± SD; *n* = 52.

4 Mixed model-based estimates presented as least square mean (95% CI).

5 Before starting period 1.

6 *n* = 51, outlier removal (*n* = 1).

7 *n* = 57, outlier removal (*n* = 1).

8 Significantly different (*P* < 0.05).

**TABLE 3:** Within- and between-condition mean differences for vascular measures in adults with prediabetes^1^

Outcome	Study baseline^15^	Pistachio		Within-condition difference^4^	Usual care		Within-condition difference^4^	Between-condition difference^4^
		Pre^2^	Post^3^		Pre^2^	Post^3^		
bSBP (mm Hg)	127.1 ± 10.9	127.6 ± 12.3	128.2 ± 12.5^7^	1.3 (−1.1, 3.7)	127.0 ± 11.4	128.8 ± 12.6	0.9 (−1.4, 3.4)	0.3 (−3.1, 3.7)
bDBP (mm Hg)	81.3 ± 7.3	81.6 ± 8.1	82.4 ± 9.4	−0.8 (−0.7, 2.4)	80.8 ± 8.0	82.0 ± 8.8	0.7 (−0.8, 2.3)	−0.1 (−2.8, 2.4)
cSBP (mm Hg)	117.3 ± 10.3	117.8 ± 11.8	119.1 ± 12.2	1.4 (−0.7, 3.6)	117.3 ± 10.8	118.8 ± 11.6	0.8 (−1.3, 3.0)	−0.6 (−2.4, 3.7)
cDBP (mm Hg)	82.0 ± 7.4	82.3 ± 8.1	83.2 ± 9.5	0.9 (−0.6, 2.5)	81.6 ± 8.1	82.8 ± 8.6	0.7 (−0.8, 2.4)	0.1 (−2.1, 2.4)
PP (mm Hg)	35.1 ± 5.7	35.5 ± 6.2	35.8 ± 5.7	0.4 (−0.9, 1.7)	35.0 ± 5.3^5^	35.8 ± 6.1	0.1 (−1.2, 1.5)	0.2 (−1.1, 1.6)
AP (mm Hg)	10.4 ± 5.8^13^	10.7 ± 5.9^6^	10.2 ± 4.4	−0.4 (−1.5, 0.5)	10.3 ± 5.8^6^	10.0 ± 4.8	−0.5 (−1.6, 0.5)	0.0 (−0.8, 1.0)
AIx^14^ (%)	23.2 ± 11.9	23.8 ± 12.6	24.5 ± 10.7	0.3 (−1.6, 2.3)	24.6 ± 11.6^5^	23.8 ± 11.4	−0.5 (−2.5, 1.5)	0.8 (−1.9. 3.7)
HR (bpm)	62.5 ± 8.5^10^	63.3 ± 8.8^12^	63.7 ± 11.1^11^	−0.1 (−1.7, 1.4)^10^	61.9 ± 9.5^5^	63.4 ± 10.5	0.3 (−1.1, 1.9)^10^	−0.5 (−2.7, 1.6)^10^
PTT (ms)	62.5 ± 7.5^10^	62.4 ± 7.4^12^	63.7 ± 8.6^11^	0.9 (−0.5, 2.5)	63.9 ± 8.5	63.7 ± 8.6	−0.1 (−1.6, 1.4)	−1.0 (−1.1, 3.2)
PWV (m/s)	8.3 ± 1.2	8.3 ± 1.2^12^	8.2 ± 1.2^8^	−0.1 (−0.3, 0.1)^10^	8.1 ± 1.2^5^	8.2 ± 1.2^7^	0.0 (−0.1, 0.3)^10^	−0.2 (−0.4, 0.0)^10^

Abbreviations: AIx, augmentation index; AP, augmentation pressure; bDBP, brachial diastolic blood pressure; bSBP, brachial systolic blood pressure; cDBP, central diastolic blood pressure; CI, confidence interval; cSBP, central systolic blood pressure; HR, heart rate; PP, pulse pressure; PTT, pulse transit time; PWV, pulse wave velocity.

^9^*n* = 49, outlier removal (*n* = 1).

^16^Significantly different (*P* < 0.05).

1 *n* = 64 unless otherwise stated. Statistical analyses were performed with SAS version 9.4 (SAS Institute). The MIXED procedure was used to determine the within- and the between-condition mean difference adjusted for the precondition value. Usual care is defined as education to consume 1–2 carbohydrate exchanges (15–30 g CHOs) as a nighttime snack.

2 Values are arithmetic means ± SD; *n* = 58.

3 Values are arithmetic mean ± SD; *n* = 52.

4 Mixed model-based estimates presented as least square mean (95% CI).

5 *n* = 57, outlier removal (*n* = 1).

6 *n* = 56, outlier removal (*n* = 2).

7 *n* = 51, outlier removal (*n* = 1).

8 *n* = 50, outlier removal (*n* = 1), missing data (*n* = 1).

10 *n* = 63, outlier removal (*n* = 1).

11 *n* = 51, missing data (*n* = 1).

12 *n* = 56, outlier removal (*n* = 1); missing data (*n* = 1).

13 *n* = 61, outliers removed (*n* = 3).

14 Adjusted to heart rate of 75 bpm.

15 Before starting period 1.

The total HEI-2015 score was 6.8 points (95% CI: 1.5, 12.1) higher after the pistachio condition than after the usual care condition (Table 4). Compared with the usual care condition, higher scores were observed after the pistachio condition for the following HEI-2015 components: seafood and plant protein [2.0 points (95% CI: 1.0, 2.9)], fatty acid ratio [1.7 points (95% CI: 0.0, 3.5)], and refined grains [2.3 points (95% CI: 1.1, 3.5)]. No other between-condition differences were observed for the HEI-2015 components. The mean HEI-2015 score for nighttime snacks before diet period 1 was 42.65 points (Supplemental Table 2). Participants were more likely to have higher HEI-2015 scores after the pistachio condition than after the usual care condition (*P* < 0.001).

**TABLE 4:** Within- and between-condition mean differences for the Healthy Eating Index-2015 score and individual components in adults with prediabetes^1^

Component	Maximum score	Study baseline^5^	Pistachio		Within-condition difference^4^	Usual care		Within-condition difference^4^	Between-condition difference^4^
			Pre^2^ (*n* = 56)	Post^3^ (*n* = 43)		Pre^2^ (*n* = 49)	Post^3^ (*n* = 46)		
Adequacy									
Total fruits	5	1.7 ± 2.0	1.7 ± 2.0	2.1 ± 2.2	0.3 (−0.2, 0.9)	1.4 ± 1.7	2.0 ± 2.1	0.7 (0.1, 1.3)^6^	−0.4 (−1.3, 0.4)
Whole fruits	5	1.9 ± 2.2	2.0 ± 2.3	2.1 ± 2.3	0.1 (−0.5, 0.7)	1.7 ± 2.1	2.2 ± 2.4	0.5 (−0.1, 1.2)	−0.4 (−1.4, 0.5)
Total vegetables	5	3.5 ± 1.6	3.4 ± 1.7	3.5 ± 1.5	0.1 (−0.3, 0.6)	4.0 ± 1.4	3.4 ± 1.6	−0.1 (−0.6, 0.3)	0.3 (−0.3, 1.0)
Greens and beans	5	2.3 ± 2.3	2.3 ± 2.3	2.6 ± 2.4	0.4 (−0.3, 1.1)	2.5 ± 2.4	2.7 ± 2.3	0.3 (−0.3, 1.1)	0.0 (−1.0, 1.0)
Whole grains	10	3.1 ± 3.7	2.6 ± 3.3	2.7 ± 3.5	−0.3 (−1.4, 0.7)	3.6 ± 3.7	2.7 ± 3.4	−0.7 (−1.8, 0.3)	0.3 (−1.2, 1.9)
Dairy	10	5.4 ± 3.6	5.5 ± 3.2	5.4 ± 3.0	−0.2 (−1.2, 0.6)	6.2 ± 3.2	5.4 ± 3.3	−0.1 (−1.0, 0.8)	−0.1 (−1.5, 1.2)
Total protein foods	5	4.2 ± 1.4	4.1 ± 1.5	4.7 ± 0.8	0.4 (0.2, 0.7)^6^	4.1 ± 1.5	4.1 ± 1.3	0.0 (−0.3, 0.4)	0.4 (−0.0, 0.8)
Seafood and plant proteins	5	1.9 ± 2.2	2.2 ± 2.3	3.9 ± 1.9	1.7 (1.0, 2.4)^6^	2.0 ± 2.3	1.9 ± 2.3	−0.2 (−0.9, 0.4)	2.0 (1.0, 2.9)^6^
Fatty acids	10	4.6 ± 3.6	4.6 ± 3.6	6.2 ± 3.8	1.9 (0.7, 3.2)^6^	3.9 ± 3.5	4.8 ± 3.8	0.1 (−1.0, 1.4)	1.7 (0.0, 3.5)^6^
Moderation									
Refined grains	10	6.3 ± 3.7	6.0 ± 3.9	7.3 ± 3.1	1.2 (0.1, 2.3)^6^	6.3 ± 3.7	5.2 ± 3.8	−1.1 (−2.1, −0.0)^6^	2.3 (1.1, 3.5)^6^
Sodium	10	3.2 ± 3.7	3.2 ± 3.6	3.5 ± 3.3	0.7 (−0.2, 1.8)	2.6 ± 3.2	2.9 ± 3.3	−0.0 (−1.1, 1.0)	0.8 (−0.6, 2.3)
Added sugars	10	8.4 ± 2.6	8.1 ± 2.6	8.7 ± 2.5	0.3 (−0.3, 1.0)	8.7 ± 2.0	8.6 ± 1.7	−0.0 (−0.7, 0.6)	0.3 (−0.6, 1.3)
Saturated fatty acids	10	5.2 ± 4.0	4.9 ± 3.9	4.8 ± 3.6	0.2 (−0.8, 1.3)	4.3 ± 3.9	5.1 ± 3.5	0.6 (−0.4, 1.7)	−0.3 (−1.8, 1.0)
Total score	100	52.3 ± 14.7	51.2 ± 14.6	58.2 ± 12.8	7.2 (3.5, 10.9)^6^	51.9 ± 15.2	51.5 ± 12.5	0.4 (−3.3, 4.1)	6.8 (1.5, 12.1)^6^

1 *n* = 61 unless otherwise stated. 24-h recalls were not completed by 3 participants. Statistical analyses were performed with SAS version 9.4 (SAS Institute). The MIXED procedure was used to determine the within- and between-condition mean difference adjusted for the precondition value. Usual care is defined as education to consume 1–2 carbohydrate (CHO) exchanges (15–30 g CHOs) as a nighttime snack.

2 Values are arithmetic means ± SD. For the pistachio condition, there were *n* = 2 missing recalls. For the usual care condition, there were *n* = 9 missing recalls.

3 Values are arithmetic mean ± SD. For the pistachio condition, missing recalls (*n* = 9) and participants withdrew from the study (*n* = 6). For the usual care condition, missing recalls (*n* = 6) and participants withdrew from the study (*n* = 6).

4 Mixed model-based estimates presented as least square mean (95% CI).

5 Before starting period 1. *n* = 57, *n* = 7 missing recalls at baseline (*n* = 2 withdrew from the study).

6 Significantly different (*P* < 0.05).

No difference in total energy was observed between the conditions (Table 5). The percentage of total energy (% kcal) from MUFA [2.0% (95% CI: 0.2, 3.8)] and potassium intake [463 mg (95% CI: 5, 920)] were higher after the pistachio condition than after the usual care condition. Fiber [4.6 g (95% CI: 0.8, 8.4)] intake was higher after the pistachio condition than after the usual care condition. Consumption of refined grains was significantly lower after the pistachio condition [−1.7 oz-eq (95% CI: −3.0, −0.3)] than after the usual care condition (Supplemental Table 3). Nut and seed intake was higher [1.8 oz-eq (95% CI: 1.1, 2.6)] after the pistachio condition than after the usual care condition. In addition, oil consumption was higher after the pistachio condition [10.9 g (95% CI: 0.2, 21.7)] than after usual care.

**TABLE 5:** Within- and between-condition mean differences for daily nutrient intake in adults with prediabetes^1^

Component	Baseline period 1^5^	Pistachio		Within-condition difference^4^	Usual care		Within-condition difference^4^	Between-condition difference^4^
		Pre (*n* = 56)^2^	Post (*n* = 43)^3^		Pre (*n* = 49)^2^	Post (*n* = 46)^3^		
Energy (kcal)	2150 ± 895	2068 ± 758	2152 ± 730	16 (−210, 244)	2241 ± 947	2141 ± 831	−71 (−301, 158)	88 (−235, 413)
Protein (g)	95.2 ± 47.6	89.5 ± 34.6	99.6 ± 43.4	4.6 (−8.2, 17.5)	102.8 ± 50.3	95.9 ± 45.1	−3.9 (−17.0, 9.1)	8.6 (−9.8, 27.0)
Protein (% kcal)	18.3 ± 6.9	18.0 ± 5.9	18.4 ± 4.7	0.1 (−1.6, 1.8)	18.9 ± 7.5	18.4 ± 6.5	0.1 (−1.6, 1.9)	−0.0 (−2.5, 2.4)
Carbohydrate (g)	230 ± 105	227 ± 101	217 ± 85	−10.1 (−38.0, 17.7)	235 ± 100	229 ± 105	−1.7 (−29.9, 26.5)	−8.4 (−48.3, 31.4)
Carbohydrate (% kcal)	43.7 ± 12.5	44.2 ± 11.8	40.7 ± 10.3	−2.3 (−5.9, 1.2)	42.9 ± 11.5	43.5 ± 13.0	0.5 (−3.0, 4.1)	−2.8 (−7.9, 2.2)
Total fat (g)	91.5 ± 55.2	88.4 ± 41.5	98.0 ± 41.4	4.4 (−8.1, 17.1)	97.2 ± 58.3	91.0 ± 51.4	−8.0 (−20.8, 4.7)	12.4 (−5.5, 30.5)
Total fat (% kcal)	37.3 ± 11.0	37.8 ± 10.5	40.8 ± 9.4	2.0 (−0.7, 4.9)	37.8 ± 9.3	37.7 ± 9.7	−1.0 (−3.9, 1.8)	3.1 (−0.9, 7.2)
SFA (g)	31.2 ± 23.6	28.9 ± 14.8	29.2 ± 14.0	−1.6 (−6.3, 2.9)	34.8 ± 25.9	29.8 ± 20.6	−4.2 (−9.0, 0.4)	2.5 (−4.1, 9.3)
SFA (% kcal)	12.4 ± 5.5	12.4 ± 4.7	12.3 ± 4.2	−0.6 (−1.9, 0.6)	13.2 ± 5.0	12.2 ± 4.3	−0.9 (−2.2, 0.4)	0.2 (−1.6, 2.0)
MUFA (g)	32.9 ± 22.4	31.6 ± 17.2	37.2 ± 19.8	3.5 (−2.1, 9.2)	34.2 ± 22.3	32.6 ± 21.9	−3.0 (−8.8, 2.8)	6.5 (−1.6, 14.7)
MUFA (% kcal)	13.2 ± 5.0	13.4 ± 4.6	15.1 ± 4.2	1.4 (0.1, 2.6)^6^	13.0 ± 3.9	13.1 ± 4.2	−0.6 (−1.9, 0.6)	2.0 (0.2, 3.8)^6^
PUFA (g)	19.3 ± 9.8	20.1 ± 11.7	22.3 ± 11.2	2.7 (−0.8, 6.3)	20.0 ± 9.9	20.8 ± 11.7	−0.3 (−3.9, 3.2)	3.0 (−2.0, 8.1)
PUFA (% kcal)	8.2 ± 3.3	8.6 ± 3.7	9.7 ± 3.8	1.3 (0.0, 2.6)^6^	8.1 ± 2.6	9.0 ± 3.9	0.4 (−0.8, 1.6)	0.9 (−0.8, 2.7)
Fiber (g)	19.6 ± 9.5	19.2 ± 10.2	22.3 ± 13.0	2.6 (−0.6, 5.9)	20.6 ± 10.3	18.8 ± 11.0	−1.9 (−5.3, 1.3)	4.6 (0.8, 8.4)^6^
Potassium (mg)	3009 ± 1300	2928 ± 1238	3106 ± 1390	116 (−204, 436)	3214 ± 1499	2790 ± 1108	−346 (−671, −22)^6^	463 (5, 920)^6^
Sodium (mg)	3789 ± 1711	3644 ± 1538	3771 ± 1531	−96 (−556, 363)	4162 ± 1731	4103 ± 1850	−19 (−485, 446)	−76 (−736, 583)

1 *n* = 61 unless otherwise stated. 24-h recalls were not completed by 3 participants. Statistical analyses were performed with SAS version 9.4 (SAS Institute). The MIXED procedure was used to determine within- and between-condition mean difference adjusted for the precondition value. Usual care is defined as education to consume 1–2 carbohydrate (CHO) exchanges (15–30 g CHOs) as a nighttime snack.

2 Values are arithmetic means ± SD. For the pistachio condition, there were *n* = 2 missing recalls. For the usual care condition, there were *n* = 9 missing recalls.

3 Values are arithmetic mean ± SD. For the pistachio condition, recalls missing (*n* = 9) and participants withdrew from the study (*n* = 6). For the usual care condition, missing recalls (*n* = 6) and participants withdrew from the study (*n* = 6).

4 Mixed model-based estimates presented as least square mean ± 95% CI.

5 Before starting period 1. *n* = 57, *n* = 7 missing recalls at baseline (*n* = 2 withdrew from the study).

6 Significantly different (*P* < 0.05).

## Discussion

In this study, consuming 57 g/d of unsalted pistachios as a nighttime snack for 12 wk did not improve FPG compared with education to consume a nighttime snack with 15–30 g of CHOs in adults with prediabetes. Similarly, improvements in insulin, HOMA-IR, lipids/lipoproteins, and vascular health were not observed with pistachio intake compared with CHO-containing snack intake. However, intake of pistachios as a nighttime snack improved diet quality, assessed by the HEI-2015, compared with intake of a CHO-containing snack. The increase in HEI-2015 was driven by improvements in the seafood and plant protein, fatty acid ratio, and refined grain components. Taken together, intake of 57 g/d of pistachios as a nighttime snack, compared with a snack containing 15–30 g of CHO, did not improve glycemic control, lipids/lipoproteins, or vascular health measures, but resulted in greater diet quality over 12 wk.

The primary hypothesis tested in this study was that intake of pistachios as a nighttime snack would lower FPG compared with education to consume a snack with 15–30 g of CHOs. This was based on prior evidence showing improved glycemic control (for example, FPG, insulin, HbA1c) with pistachio intake [32,36] in adults with impaired glycemia and current clinical practices around nighttime snacking [7,10]. Several meta-analyses show pistachios consistently improve glycemic control when compared with no nut controls in healthy adults and those with CVD and/or T2DM risk factors [[16], [17], [18], [19], [20]]. However, effect estimates tend to be modest for fasting glucose (<8 mg/dL), insulin (<5 μIU/mL), HOMA-IR (<3 units), and HbA1c (<0.6%), which likely cannot be detected in trials with relatively small sample sizes. This trial was powered to detect a 10 mg/dL difference in FPG, and a small effect was observed [difference: 0.9 mg/dL (95% CI: −1.2, 3.1); Cohen’s *d* effect size: 0.18]. On the basis of Cohen’s *d* effect size and the bounds of the 95% CI, it is unlikely that a clinically relevant effect remained undetected because of a lack of statistical power. Therefore, our results show that intake of 57 g/d of pistachios as a nighttime snack is not more effective than a common clinical approach of education to consume a snack containing 1–2 exchanges of CHO for reducing fasting glucose. However, pistachios did not worsen FPG compared with CHO counting and therefore may be an alternative nighttime snack for individuals who prefer pistachios to CHO-containing snacks.

Our study also did not demonstrate between-condition differences in lipids/lipoproteins, BP, and vascular health. These effects are incongruent with a network meta-analysis of 34 clinical trials that showed pistachio-enriched diets were the best ranked among walnut-, hazelnut-, and cashew-containing diets forlowering total cholesterol (−5%), LDL cholesterol (−5%), and triacylglycerols (TAGs; −10%) in healthy adults or those with CVD risk factors [41]. However, a recent meta-analysis of 17 RCTs showed no improvement in SBP, DBP, TAG, and HDL cholesterol with pistachio intake among individuals with prediabetes despite significant effects for SBP (MD: −2.89 mmHg), TAGs (MD: −16.76 mg/dL), and HDL cholesterol (MD: 1.43 mg/dL) in the pooled analysis including healthy individuals and those with prediabetes, metabolic syndrome, and T2DM [20]. Differences in effects may be explained by the degree of metabolic dysfunction present in the study population. Our trial included individuals who met the ADA criteria for prediabetes but were otherwise generally healthy. Moreover, average glucose impairment at the beginning of the trial in our cohort (102.5 mg/dL) was only slightly above the cut point for impaired FPG (≥100 mg/dL) and mean HbA1c was within the normal range (5.4%). More intensive dietary interventions at multiple timepoints across the day may be necessary to improve risk markers at the onset of metabolic dysfunction.

The results of this trial may have been influenced by weight changes during the pistachio condition. After the pistachio condition, there was a small increase in weight [0.7 kg (95% CI: 0.1, 1.4)] and BMI [0.2 kg/m^2^ (95% CI: 0.0, 0.4)] that was not observed during the usual care condition; however, there was no significant between-condition difference in weight [42]. A meta-analysis of 33 RCTs showed that nut consumption did not increase weight when nuts replace other foods in the diet or when added to the usual diet [42]. Effects of pistachio intake on weight are not consistent with some meta-analyses of RCTs showing no effect on weight [43] or an increase in weight [Weighted mean difference: 0.19 kg (95 % CI: 0.12, 0.26)] [16]. Increased BMI [0.6 kg/m^2^ (95%: 0.2, 1.0)] was observed in a crossover trial in which 50 g/d of almonds were provided to consume as part of habitual intake compared with education to consume a diet consistent with Dutch dietary guidelines over 5 months [44]. Whole body insulin sensitivity was lower after the almond condition than after the control but including BMI as a covariate in the mixed-models only partly explained the lower insulin sensitivity. The inconsistent effects of pistachios and nut intake on weight is likely explained by intervention delivery and implementation. In this trial, participants were asked to consume the pistachios after dinner but before bed and replace current nighttime snacks if consumed. Study foods were not expected to increase net energy intake considering the average adult in the United States consumes ∼370 kcal/d as snacks based on data from the 2013–2016 NHANES survey [45]. We did not provide any instructions about energy intake at other snack or mealtimes. This approach may have led to unexpected dietary modifications such as consuming typical nighttime snacks (for example, ice cream) at meals or other snacking periods. The slight weight gain during the pistachio condition suggests that the pistachio snack added to daily energy intake despite instructions to incorporate the snack in place of foods usually consumed during the evening. More explicit instructions that included education about intake of foods at other times of the day may have been needed to ensure energy balance and avoid weight gain.

Our results show that intake of pistachios as a nighttime snack may be an effective strategy to improve dietary alignment with the Dietary Guidelines for Americans. The HEI-2015 score was ∼7 points higher after the pistachio condition than after the usual care condition. In addition, the pistachio condition was more likely to improve the HEI-2015 scores of nighttime snacks compared with education to consume 1–2 CHO exchanges. However, the improvement in the HEI-2015 did not translate to beneficial shifts in chronic disease risk factors. A 5–6 point increase in the HEI is associated with lower risk of diet-related chronic diseases and is considered meaningful [46]. Similar increases in diet quality scores have been shown to improve CVD risk factors in clinical trials, but many involve weight loss interventions where multiple food behaviors were the focus and comparator conditions were no-treatment controls [47,48]. Although CVD risk factors were expected to improve in this trial because of the improvement in diet quality with the pistachio intervention, HEI-2015 scores were low at baseline and remained poor (<59 points) with the pistachio intervention. Manipulating the quality of the nighttime snack for individuals with impaired FPG and very low baseline diet quality may not be sufficient to elicit diet quality-related improvements in CVD risk factors within a 12-wk period.

The strengths of this study are the design, measures of short- and longer-term glycemic control, and method of dietary assessment. The crossover design included baseline measures for each condition allowing for change from baseline estimates for MDs between conditions. Measures of fasting glycemic control are corroborated by the longer-term measures of glycemic control including HbA1c. Measures of diet quality and nutrient composition were collected using the validated ASA24, which allows for the identification of differences in dietary intake between conditions. However, this trial has several limitations. The COVID-19 pandemic resulted in trial suspension and the withdrawal of 13 participants, which contributed to a large attrition rate and missing data. In addition, the age of inclusion criterion had to be modified as part of the University approval process to restart the study, which limits the generalizability of the findings to individuals aged 65–75 y. The study protocol was single blinded because the nature of the trial did not allow for blinding of participants. Although single blinding may introduce bias, outcome assessors were blinded for the data collection period. A single nonrandom 24-h recall was conducted at the beginning and end of each diet period, which does not capture individual level day-to-day variation in intake and has the potential for recall bias. Finally, there is inflated risk of type I error for secondary endpoints because this trial is likely too small to meet the rigor of correction methods [49]. Significant findings among secondary endpoints are hypothesis-generating.

In summary, this trial showed that 57 g/d of pistachios as a nighttime snack did not lower FPG compared with education to consume a nighttime snack with 15–30 g of CHO, but improved diet quality. Pistachios may be a convenient nighttime snacking option to improve adherence to the Dietary Guidelines for Americans for those with prediabetes. Additional research is needed to identify convenient dietary strategies that may be employed at early stages to delay or reverse impaired glycemic control.

## Acknowledgments

We thank Marcella Smith for assistance with food preparation and education provided to participants. In addition, thank you to the nursing staff at the Penn State Clinical Research Center for their assistance with data collection.

### Author contributions

The authors’ responsibilities were as follows – KSP, PMK-E: designed the research; TMR, TLH: conducted the research; TMR, KSP: analyzed and interpreted the data; TMR: drafted the manuscript; PMK-E, TLH, KSP: critically reviewed the manuscript; KSP: had primary responsibility for final content; and all authors: read and approved the final manuscript.

### Conflict of interest

KSP and PMK-E received a grant to conduct the research from The American Pistachio Growers. The other authors report no conflict of interest.

### Funding

This trial was funded by the American Pistachio Growers. The American Pistachio growers had no role in study design, data collection, data analysis, data interpretation, or writing of the manuscript. The research conducted was supported by the National Center for Advancing Translational Sciences, National Institutes of Health, through Grant UL1 TR002014. The content is solely the responsibility of the authors and does not necessarily represent the official views of the NIH.

### Data availability

Data described in the manuscript, code book, and analytic code will be made available upon request pending application and approval.

## Appendix A Supplementary data

Multimedia component1 Multimedia component1
