# Supplementary material for: Intake of Pistachios as a Nighttime Snack Has Similar Effects on Short- and Longer-Term Glycemic Control Compared with Education to Consume 1–2 Carbohydrate Exchanges in Adults with Prediabetes: A 12-Wk Randomized Crossover Trial
Source: J Nutr. 2024 Jan 24;154(4):1219–31. doi: 10.1016/j.tjnut.2024.01.021 (PMC11347794; doi:10.1016/j.tjnut.2024.01.021)
Supplement: Multimedia component1 [file mmc1.docx]

**Intake of pistachios as a nighttime snack has similar effects on short and longer-term glycemic control compared to education to consume 1-2 carbohydrate exchanges in adults with prediabetes: A 12-week randomized crossover trial**

Terrence M. Riley PhD, RD

# Supplementary Materials

## Table S1 Number of participants available for analysis before and after outliers were removed for each condition at baseline and endpoint

| Variable |  | Pistachio | | Usual Care | |
| --- | --- | --- | --- | --- | --- |
|  |  | Pre | Post | Pre | Post |
| Glucose | total | 58 | 52 | 58 | 52 |
|  | total after outliers | 58 | 51 | 58 | 52 |
| Insulin | total | 58 | 52 | 58 | 52 |
|  | total after outliers | 58 | 52 | 58 | 52 |
| HbA1c | total | 58 | 52 | 58 | 52 |
|  | total after outliers | 58 | 52 | 58 | 52 |
| HOMA-IR | total | 58 | 52 | 58 | 52 |
|  | total after outliers | 58 | 51 | 58 | 52 |
| TC | total | 58 | 52 | 58 | 52 |
|  | total after outliers | 58 | 52 | 58 | 52 |
| LDL-C | total | 58 | 52 | 58 | 52 |
|  | total after outliers | 58 | 52 | 58 | 52 |
| HDL-C | total | 58 | 52 | 58 | 52 |
|  | total after outliers | 58 | 52 | 58 | 52 |
| TAG | total | 58 | 52 | 58 | 52 |
|  | total after outliers | 58 | 52 | 57 | 52 |
| SBP | total | 58 | 52 | 58 | 52 |
|  | total after outliers | 58 | 51 | 58 | 52 |
| DBP | total | 58 | 52 | 58 | 52 |
|  | total after outliers | 58 | 52 | 58 | 52 |
| Central SBP | total | 58 | 52 | 58 | 52 |
|  | total after outliers | 58 | 51 | 58 | 52 |
| Central DBP | total | 58 | 52 | 58 | 52 |
|  | total after outliers | 58 | 52 | 58 | 52 |
| PP | total | 58 | 52 | 58 | 52 |
|  | total after outliers | 58 | 52 | 57 | 52 |
| AP | total | 58 | 52 | 58 | 52 |
|  | total after outliers | 56 | 52 | 56 | 52 |
| AIx | total | 58 | 52 | 58 | 52 |
|  | total after outliers | 58 | 52 | 57 | 52 |
| HR | total | 58 | 52 | 58 | 52 |
|  | total after outliers | 56 | 51 | 57 | 52 |
| PTT | total | 57 | 51 | 58 | 52 |
|  | total after outliers | 56 | 51 | 58 | 52 |
| PWV | total | 57 | 51 | 58 | 52 |
|  | total after outliers | 56 | 50 | 57 | 51 |

AIx, Augmentation index; AP, Augmentation pressure; DBP, Diastolic blood pressure; HbA1c, Hemoglobin A1c; HDL-C, high-density lipoprotein-cholesterol; HOMA-IR, Homeostatic assessment model of insulin resistance; HR, Heart rate; LDL-C, Low-density lipoprotein cholesterol; PP, pulse pressure; PTT, pulse transit time; PWV, pulse wave velocity; SBP, Systolic blood pressure; TAG, triglyceride.

## Table S2 Likelihood that HEI-2015 scores were higher or lower than

**HEI-2015 scores at the beginning of the trial^1^**

|  |  | Usual Care | |
| --- | --- | --- | --- |
|  |  | Higher | Lower |
| Pistachio | Higher | 8 (29.6) | 17 (62.9) |
|  | Lower | 0 (0) | 2 (7.4) |
| ^1^n = 27. Values are count (% total). McNemar’s exact test (PROC FREQ) was used to compare the likelihood that nighttime snacks reported at the end of conditions were higher or lower than nighttime snacks prior to the beginning of the trial. Data include only those who reported nighttime snacks at the first diet recall and at the end of each condition. Chi^2^ = 17.00; DF = 1; Exact p < 0.001. | | | |

## Table S3 Within- and between-condition mean differences for food group intake in adults with prediabetes^1^

| Component | Study Baseline ^5^ | Pistachio | | Within-condition difference^4^ | Usual care | | Within-condition difference^4^ | Between-condition difference^4^ |
| --- | --- | --- | --- | --- | --- | --- | --- | --- |
|  |  | Pre (n = 56)^2^ | Post (n = 43)^3^ |  | Pre (n = 49)^2^ | Post (n = 46)^3^ |  |  |
| Total vegetables, c-eq | 2.3 ± 1.8 | 2.1 ± 1.5 | 2.0 ± 2.6 | -0.1 (-0.5, 0.3) | 2.7 ± 2.4 | 1.9 ± 1.7 | -0.6 (-1.0, -0.1)* | 0.5 (-0.1, 1.1) |
| Dark vegetables, c-eq | 0.4 ± 0.8 | 0.4 ± 0.8 | 0.4 ± 0.7 | -0.1 (-0.3, 0.1) | 0.5 ± 1.0 | 0.3 ± 0.5 | -0.0 (-0.2, 0.0) | -0.0 (-0.2, 0.2) |
| Red/Orange vegetables, c-eq | 0.5 ± 0.5 | 0.5 ± 0.5 | 0.4 ± 0.7 | -0.1 (-0.3, 0.1) | 0.5 ± 0.5 | 0.4 ± 0.3 | -0.1 (-0.26, -0.0)* | 0.0 (-0.2, 0.2) |
| Starchy vegetables, c-eq | 0.5 ± 0.8 | 0.3 ± 0.5 | 0.4 ± 0.8 | 0.0 (-0.2, 0.2) | 0.7 ± 1.1 | 0.4 ± 0.5 | -0.0 (-0.3, 0.1) | 0.1 (-0.2, 0.4) |
| Total grains, oz-eq | 6.4 ± 3.7 | 6.4 ± 3.7 | 5.9 ± 3.1 | -0.9 (-1.9, 0.0) | 7.0 ± 4.3 | 7.4 ± 5.1 | 0.7 (-0.8, 2.3) | -1.7 (-3.4, 0.0) |
| Whole grains, oz-eq | 1.1 ± 1.6 | 0.9 ± 1.5 | 0.9 ± 1.5 | -0.2 (-0.7, 0.2) | 1.3 ± 1.7 | 1.0 ± 1.6 | -0.3 (-0.8, 0.1) | 0.0 (-0.6, 0.7) |
| Refined grains, oz-eq | 5.2 ± 3.2 | 5.4 ± 3.7 | 4.9 ± 3.0 | -0.6 (-1.5, 0.3) | 5.7 ± 4.0 | 6.4 ± 4.3 | 1.0 (-0.2, 2.4) | -1.7 (-3.0, -0.3)* |
| Total fruit, c-eq | 0.8 ± 1.2 | 0.9 ± 1.5 | 0.8 ± 1.0 | -0.0 (-0.3, 0.2) | 0.6 ± 1.0 | 0.9 ± 1.1 | 0.1 (-0.1, 0.5) | -0.2 (-0.7, 0.2) |
| Total dairy products, c-eq | 1.9 ± 2.0 | 1.6 ± 1.3 | 1.6 ± 1.4 | -0.1 (-0.5, 0.2) | 2.2 ± 2.0 | 1.5 ± 1.2 | -0.4 (-0.7, -0.0)* | 0.2 (-0.3, 0.7) |
| Total protein foods, oz-eq | 6.9 ± 4.7 | 6.9 ± 4.6 | 9.3 ± 4.6 | 2.2 (0.5, 3.8)* | 6.8 ± 4.8 | 7.2 ± 5.6 | -0.1 (-1.7, 1.5) | 2.3 (-0.0, 4.6) |
| Meat, poultry and seafood, oz-eq | 5.0 ± 4.1 | 4.8 ± 3.8 | 6.1 ± 4.5 | 0.8 (-0.7, 2.3) | 5.6 ± 4.6 | 5.8 ± 4.8 | 0.5 (-1.0, 2.1) | 0.2 (-1.9, 2.5) |
| Beef, veal, pork and lamb, oz-eq | 2.2 ± 3.3 | 1.5 ± 2.1 | 2.6 ± 3.9 | 0.2 (-1.0, 1.5) | 3.1 ± 4.0 | 2.9 ± 3.8 | 0.5 (-0.7, 1.9) | -0.3 (-2.2, 1.6) |
| Cured meats, oz-eq | 0.8 ± 1.5 | 1.0 ± 1.9 | 1.4 ± 2.3 | 0.3 (-0.4, 1.1) | 0.8 ± 1.9 | 1.1 ± 2.3 | 0.2 (-0.5, 1.0) | 0.1 (-0.9, 1.2) |
| Poultry, oz-eq | 1.1 ± 2.1 | 1.4 ± 2.0 | 1.4 ± 2.2 | 0.3 (-0.3, 1.0) | 0.8 ± 1.9 | 1.4 ± 2.4 | 0.1 (-0.6, 0.8) | 0.2 (-0.8, 1.2) |
| Seafood (high n-3), oz-eq | 0.3 ± 1.5 | 0.3 ± 2.1 | 0.0 ± 0.2 | -0.4 (-0.5, -0.3)* | 0.3 ± 1.3 | 0.3 ± 1.5 | -0.1 (-0.6, 0.3) | -0.2 (-0.7, 0.2) |
| Seafood (low n-3), oz-eq | 0.4 ± 1.5 | 0.4 ± 1.6 | 0.5 ± 1.7 | 0.2 (-0.1, 0.6) | 0.3 ± 1.3 | 0.0 ± 0.2 | -0.2 (-0.7, 0.1) | 0.5 (-0.0, 1.1) |
| Eggs, oz-eq | 0.7 ± 0.9 | 0.8 ± 1.0 | 0.5 ± 0.8 | -0.2 (-0.4, 0.0) | 0.5 ± 0.6 | 0.5 ± 0.8 | -0.1 (-0.4, 0.0) | -0.0 (-0.2, 0.2) |
| Soy, oz-eq | 0.1 ± 0.5 | 0.1 ± 0.5 | 0.2 ± 0.8 | 0.0 (-0.1, 0.3) | 0.1 ± 0.6 | 0.1 ± 0.4 | -0.0 (-0.1, 0.1) | 0.1 (-0.0, 0.3) |
| Nuts and Seeds, oz-eq | 0.9 ± 3.3 | 1.1 ± 3.4 | 2.4 ± 2.0 | 1.3 (0.7, 1.9)* | 0.4 ± 1.1 | 0.7 ± 2.5 | -0.5 (-0.9, -0.1)* | 1.8 (1.1, 2.6)* |
| Legumes, oz-eq | 0.4 ± 1.2 | 0.3 ± 1.2 | 0.5 ± 0.2 | 0.1 (-0.3, 0.6) | 0.4 ± 1.3 | 0.4 ± 1.0 | 0.0 (-0.2, 0.4) | 0.0 (-0.4, 0.6) |
| Oils^6^, g | 30.7 ± 25.6 | 31.8 ± 26.8 | 38.9 ± 26.8 | 6.6 (-0.9, 14.2) | 30.1 ± 22.1 | 29.6 ± 22.5 | -4.3 (-12.0, 3.3) | 10.9 (0.2, 21.7)* |
| ^1^n = 61 unless otherwise stated. 24 hrs recall was not completed by 3 participants. Baseline diet data was missing for 3 participants. Statistical analyses were performed with SAS version 9.4 (SAS Institute). The MIXED procedure was used to determine within- and between- condition mean difference adjusted for the pre-condition value. Usual care is defined as education to consume 1-2 carbohydrate (CHO) exchanges each night (15 g - 30 g CHO). c-eq, cup-equivalent; oz-eq, ounce-equivalent; g, grams; SE, standard error. | | | | | | | | |
| ^2^Values are arithmetic means ± SD. For the pistachio condition, missing recalls (n = 2). For the usual care condition, missing recalls (n = 9). | | | | | | | | |
| ^3^Values are least square mean ± SD. For the pistachio condition, missing recalls (n = 9) and participants withdrew from the study (n = 6). For the usual care condition, missing recalls (n = 6) and participants withdrew from the study (n = 6). | | | | | | | | |
| ^4^Mixed model-based estimates presented as least square mean ± 95% CI | | | | | | | | |
| ^5^n = 57, n = 7 missing recalls at baseline (n = 2 withdrew from the study) | | | | | | | | |
| ^6^Includes fats naturally present in nuts, seeds, seafood; unhydrogenated vegetable oils, except palm oil, palm kernel oil, coconut oils; fat in avocado and olives above allowable amount; 50% of fat present in stick/tub margarines, margarine spreads (grams) | | | | | | | | |
| *Significantly different (p < 0.05) | | | | | | | | |
